# Fecal carriage of ESBL-producing *Escherichia coli* in Egyptian patients admitted to the Medical Research Institute hospital, Alexandria University

**DOI:** 10.3934/microbiol.2020025

**Published:** 2020-11-03

**Authors:** Amira ElBaradei, Dalia Ali Maharem, Ola Kader, Mustafa Kareem Ghareeb, Iman S. Naga

**Affiliations:** 1 Department of Microbiology and Immunology, Faculty of Pharmacy, Pharos University in Alexandria, Alexandria, Egypt; 2 Alexandria University Hospital, Alexandria University, Alexandria, Egypt; 3 Department of Internal Medicine, Medical Research Institute, Alexandria University, Alexandria, Egypt; 4 Department of Microbiology, Medical Research Institute, University of Alexandria, Alexandria, Egypt; 5 Bachelor of Medical Laboratory Technology, Al-Yarmouk University, Iraq

**Keywords:** Faecal carriage, Commensal *E.coli*, ESBL, *bla*_CTX-M_

## Abstract

Commensal ESBL-producing *E. coli* represent a reservoir for resistance genes therefore, their detection is crucial to restrain the spread of beta-lactam resistance. Hence, the aim of the present study was phenotypic and genotypic characterization of commensal ESBL-producing *E. coli* obtained from the stool of patients at the time of admission and at the time of discharge from the Medical Research Institute hospital. A total of 70 *E. coli* isolates were collected from 35 patients and were categorized into Group A (samples obtained on admission) and Group B (samples obtained at the time of discharge). Phenotypically, 30 isolates were ESBL producers (40% of *E. coli* isolates collected on admission and 45.7% of the strains obtained at the time of discharge were ESBL producers). Most of them harbored one to three plasmids with sizes ranging from one kbp to ten kbp. Upon genotypic investigation, *bla*_CTX-M_ was the most detected gene in 80% of ESBL strains, followed by *bla*_TEM_ in 53.3% and the least detected was *bla*_SHV_ in only 13.3%. By comparing group A and group B, ten patients were found to carry commensal ESBL-producing *E. coli*, in two patients these isolates carried ESBL genes that were identical on admission and on discharge. However, in eight patients, these isolates carried different ESBL genes, which were newly harbored during hospital stay. The high abundance of MDR commensal *E. coli* 48.57% together with the presence of 42.86% ESBL-producing commensal *E. coli* among our isolates represents an alarming threat, as they are frequently associated with the increased risk of infection, higher costs and longer hospital stay.

## 1. Introduction

Resistance to antimicrobial agents has become a clear and present global threat. Different antibacterial agents are rendered ineffective due to the growing number of resistant bacteria, which are constantly acquiring different resistance mechanisms. Extended-spectrum beta-lactamases (ESBL), are considered as a cornerstone in the antimicrobial resistance problem [1],[2].

Beta-lactam resistance in Gram-negative bacteria is mainly mediated by ESBL enzymes. They are encoded by genes that are often located on transferable conjugative plasmids. Additionally, genes that confer resistance to antibacterial agents, other than beta-lactams, are frequently carried simultaneously on these plasmids and contribute to the dissemination of resistance [3].

In early 1990s, ESBL-producing Gram-negative bacteria used to be confined to hospitals and intensive care units (ICU), however, this soon changed with the occurrence of infections caused by ESBL-producing *E. coli* in the community [4].

Apart from infections, another reason for concern is the colonization with ESBL-producing bacteria. Commensal ESBL-producing strains represent a reservoir for resistance genes [5]. The increasing rates of carriage of ESBL-producing *E. coli* are rather challenging in healthcare settings for two reasons. The first reason is that fecal colonization by ESBL-producing *E. coli* is linked to the increased risk of invasive infections by the same strain. The second one is that this colonization contributes to the resistance gene pool, which may be acquired by susceptible bacteria. Both of which, often lead to suboptimal clinical outcomes with higher costs and longer hospital stay [6]–[8].

To contain the spread of ESBL-producing *E. coli*, implementation of infection control measures is necessary together with the detection of carriers of ESBL-producing *E. coli* [7]. Therefore, the aim of the present study was phenotypic and genotypic characterization of commensal ESBL-producing *E. coli* obtained from the stool of patients; at the time of admission and at the time of discharge from the Medical Research Institute (MRI) hospital.

## 2. Materials and methods

Stool samples were collected from 35 patients, admitted to the Internal Medicine Department of the MRI hospital. Stool samples were collected from patients at the time of admission to the hospital (Group A strains) and at the time of discharge (Group B strains). Sample collection was performed after the approval of the Ethical Committee of the MRI and after obtaining a written consent from the patients.

### 2.1. Bacterial isolation and identification

All specimens were cultured on blood and MacConkey's agar. After overnight incubation, Gram negative, lactose fermenting colonies were further identified using automated VITEK-2^®^ system (BioMérieux, France).

### 2.2. Antimicrobial susceptibility testing and phenotypic detection for ESBL

Antimicrobial susceptibility testing and screening for ESBL production was performed using VITEK-2^®^ system (BioMérieux, France), according to the manufacturer's instructions. Antibiotics used in antimicrobial susceptibility testing were: ampicillin (AMP), ceftriaxone (CRO), cefepime (FEP), aztreonam (ATM), ertapenem (ETM), imipenem (IMP), meropenem (MEM), amikacin (AK), gentamicin (GEN), tobramycin (TOB), ciprofloxacin (CIP), moxifloxacin (MFX), tigecycline (TIG) and trimethoprim-sulfamethoxazole (COT).

Then, ESBL production was confirmed according to the Clinical & Laboratory Standards Institute (CLSI) guidelines using disk-diffusion method [9]. A disc of cefotaxime (CTX) alone and cefotaxime-clavulanate (CCT) (30/10 µg) and a disc of ceftazidime (CAZ) alone and ceftazidime-clavulanate (CZA) (30/10 µg) were used. A ≥5 mm increase in a zone diameter for either antimicrobial agents in combination with clavulanate versus the zone diameter of the agent when tested alone is considered positive for ESBL production [9]. All disks used were purchased from (BioRad, USA).

### 2.3. Bacterial storage and revival

Bacterial isolates were stored at −80 °C in Luria Bertani (LB) broth tubes containing 30% glycerol [10]. For bacterial revival, one loopful was streaked over blood agar and incubated for 16–18 hours at 37 °C.

### 2.4. Genotypic detection of ESBL

ESBL-producing stains were further investigated genotypically for the presence of *bla*_CTX-M_, *bla*_SHV_ and *bla*_TEM_ genes. *bla*_SHV_ and *bla*_CTX-M_ were detected in a multiplex PCR reaction, while *bla*_TEM_ was detected using singleplex PCR. The details of the primers used, are listed in (Table 1). DNA extraction was performed by boiling method [11]. Briefly, two to three colonies from overnight colonies cultured on LB agar were suspended in TE buffer containing 0.1% triton X100. Bacterial suspensions were incubated in a boiling water bath for 15 minutes followed by rapid cooling on ice. After centrifugation for 15 minutes at 14,000 RPM in a microfuge, the supernatant was used as a source for bacterial DNA.

PCR was performed in 25 µL final volume containing 12.5 µL hot start PCR master mix MyTaq™ Red Mix (BioLine, London, UK), 10 picomoles of each primer (all primers were purchased from Thermo Fisher Scientific, California., USA) (Table 1) and 0.5 µL bacterial DNA. A negative control was prepared by the addition of the same contents to the tube with water instead of the extract. All PCR reactions were performed on Veriti thermal cycler (Applied Biosystems, California, USA).

The reactions were performed according to the following thermal profile, initial denaturation 95 °C for three minutes followed by 35 cycles of denaturation at 95 °C for 30 seconds, annealing for 30 seconds at 50 °C for *bla*_CTX-M_ and *bla*_SHV_ and at 46 °C for *bla*_TEM_, [12]–[14] then extension at 72 °C for one min/kb followed by final extension at 72 °C for 10 minutes. PCR products were separated by gel electrophoresis on two percent agarose gel containing 0.5 µg/mL ethidium bromide.

Plasmid extraction was performed on all ESBL-producing strains using Column-Pure Plasmid Miniprep Kit (Applied Biological Materials (ABM); Canada) [15]. Extracted plasmid DNA was stored at −20 °C. Plasmid DNA was then separated by gel electrophoresis on one percent agarose gel containing 0.5 µg/mL ethidium bromide. The DNA bands were visualized, on a 302 nm UV transilluminator, and photographed. The plasmid sizes were determined using standard DNA molecular weight markers at 1 kb ladder.

**Table 1.** Primers used for detection of ESBL genes in this study.

Primer	Nucleotide Sequence (5′–3′)	Annealing temperature °C	Amplicon length in bases	Reference
*bla*_CTX-M_ (F)	CGCTTTGCGATGTGCAG	50	551	[12]
*bla*_CTX-M_ (R)	ACCGCGATATCGTTGGT			
*bla*_SHV_ (F)	GCAAAACGCCGGGTTATTC	50	937	[13]
*bla*_SHV_ (R)	GGTTAGCGTTGCCAGTGCT			
*bla*_TEM_ (F)	ATGAGTATTCAACATTTCCG	46	851	[14]
*bla*_TEM_ (R)	TTAATCAGTGAGGCACCTAT			

## 3. Results

A total of 70 *E. coli* isolates were collected from 35 patients and were categorized into Group A (samples obtained from patients on admission) and Group B (samples obtained from patients at the time of discharge). The average stay of each patient in the Internal Medicine Department of the MRI hospital was three to five days.

### 3.1. Antimicrobial Susceptibility testing

Susceptibility testing of the 70 *E. coli* isolates revealed that 51.4 % of the isolates were resistant to one or more of the beta-lactams. In group A, resistance to ceftriaxone was 48.57%, which was identical to the resistance to cefepime, while in group B resistance to ceftriaxone and cefepime was 45.71% and 40%, respectively. Interestingly, all our strains in both groups were sensitive to imipenem and meropenem. The susceptibility patterns of both groups are shown in Table 2 and Table 3. By comparing group A and group B, 25 patients out of the 35 (71.4%) patients carried commensal *E. coli* with the same susceptibility pattern to CRO and FEP, while 10 (28.6%) patients carried commensal *E. coli* with different susceptibility pattern to these antibiotics.

### 3.2. Detection of ESBL production

Phenotypically, 30 isolates were ESBL producers, using automated VITEK-2^®^ system (BioMérieux, France), and the results were confirmed using ESBL test by disk-diffusion method according to CLSI guidelines. Fourteen out of the 35 (40%) *E. coli* isolates collected at time of hospital admission were ESBL producers. After hospital admission, ten of these *E. coli* isolates remained ESBL producers, while another six isolates became ESBL producers, so that the total number of ESBL- producing isolates in group B (after hospital admission) became 16 out of the 35 (45.7%). All ESBL- producing strains in both Groups A and B were MDR.

Plasmid extraction was performed for all our 30 ESBL-producing *E. coli* strains. Most of them harbored one-three plasmids with sizes ranging from one kbp to ten kbp.

Upon genotypic investigation, *bla*_CTX-M_ was the most detected gene in 80% of ESBL strains, followed by *bla*_TEM_ in 53.3% and the least detected was *bla*_SHV_ in only 13.3% (Table 4). By comparing group A and group B, ten patients were found to carry commensal ESBL-producing *E. coli*. In two patients these isolates carried ESBL genes that were identical on admission and on discharge. However, in eight patients, these isolates carried different ESBL genes that were newly harbored during hospital stay (Table 5). There was no difference in plasmid sizes between isolates obtained from the patients on admission and on discharge.

**Table 2.** Antimicrobial susceptibility pattern of the *E. coli* isolates to beta-lactams antibiotics.

	Ampicillin (AMP)		Ceftriaxone (CRO)		Cefepime (FEP)		Aztreonam (ATM)		Ertapenem (ETM)		Imipenem (IMP)		Meropenem (MEM)	
	S	R	S	R	S	R	S	R	S	R	S	R	S	R
Group A	17	18	18	17	18	17	18	17	34	1	35	0	35	0
	48.57%	51.43%	51.43%	48.57%	51.43%	48.57%	51.43%	48.57%	97.14%	2.86%	100%	0%	100%	0%
Group B	17	18	19	16	21	14	18	17	35	0	35	0	35	0
	48.57%	51.43%	54.29%	45.71%	60%	40%	51.43%	48.57%	100%	0%	100%	0%	100%	0%

**Table 3.** Antimicrobial susceptibility pattern of the *E. coli* isolates to antibiotics other than beta-lactams.

	Amikacin (AK)		gentamicin (GEN)		Tobramycin (TOB)		Ciprofloxacin (CIP)		Moxifloxacin (MFX)		Tigecycline (TIG)		Trimethoprim-sulfamethoxazole (COT)	
	S	R	S	R	S	R	S	R	S	R	S	R	S	R
Group A	35	0	35	0	25	10	24	11	24	11	35	0	26	9
	100%	0%	100%	0%	71.43%	28.57%	68.57%	31.43%	68.57%	31.43%	100%	0%	74.29%	25.71%
Group B	35	0	34	1	24	11	24	11	22	13	35	0	27	8
	100%	0%	97.14%	2.86%	68.57%	31.43%	68.57%	31.43%	62.86%	37.14%	100%	0%	77.14%	22.86%

**Table 4.** ESBL genes detected among our ESBL-producing *E. coli* isolates.

	ESBL strains among Group A (n=14)		ESBL strains among Group B (n=16)		Total (n=30)	
Genes	*N*	*%*	*N*	*%*	*N*	*%*
*bla*_CTX-M_	10	71.43%	14	87.50%	24	80%
*bla*_SHV_	3	21.43%	1	6.25%	4	13.33%
*bla*_TEM_	6	42.86%	10	62.5%	16	53.33%

**Table 5.** Characterization of the 30 ESBL-producing *E. coli* isolates.

Group A ESBL-Producing *E. coli*									Group B ESBL-Producing *E. coli*								
*E. coli* Isolate	*blaCTX-M*	*blaSHV*	*blaTEM*	AMP	CRO	FEP	ATM	N. of plasmids	*E. coli* Isolate	*blaCTX-M*	*blaSHV*	*blaTEM*	AMP	CRO	FEP	ATM	N. of plasmids
1A	_	_	_	R	R	R	R	3	1B	+	_	+	R	R	R	R	3
2A	+	_	_	R	R	R	R	1									
7A	+	_	+	R	R	R	R	3	7B	+	_	_	R	R	R	R	3
9A	+	_	_	R	R	R	R	-									
10A	+	_	_	R	R	R	R	2	10B	_	_	+	R	R	R	R	2
14A	_	_	_	R	R	R	R	1	14B	_	+	+	R	S	S	R	1
17A	_	_	_	R	R	R	R	3	17B	+	_	+	R	R	R	R	3
18A	_	+	+	R	R	R	R	1	18B	+	_	+	R	R	R	R	1
19A	+	_	_	R	R	R	R	1	19B	+	_	_	R	R	R	R	1
20A	+	+	+	R	R	R	R	2	20B	+	_	_	R	R	R	R	2
23A	+	_	_	R	R	R	R	-									
24A	+	+	+	R	R	R	R	-									
									25B	+	_	+	R	R	R	R	1
									26B	+	_	+	R	R	R	R	3
									27B	+	_	_	R	R	R	R	1
									28B	+	_	+	R	R	S	R	-
									30B	+	_	+	R	R	R	R	-
31A	+	_	+	R	R	R	R	1	31B	+	_	+	R	R	S	R	1
									32B	+	_	_	R	R	R	R	-
35A	+	_	+	R	R	R	R	1	35B	+	_	_	R	R	R	R	1

## 4. Discussion

Infections caused by ESBL-producing *E. coli* are considered a global health challenge. In fact, they represent one of the main reasons for beta-lactams therapy failure [16]. Interestingly, infections with ESBL-producing *E. coli* are, in many cases, preceded by asymptomatic carriage [8],[17],[18]. Therefore, the aim of the present study was phenotypic and genotypic characterization of commensal ESBL-producing *E. coli* obtained from the stool of patients; at the time of admission and at the time of discharge from the MRI hospital.

In the present study, resistance to the extended spectrum cephalosporins in both groups ranged from 40% to 48.57%, while resistance to aztreonam was 48.57% in both groups. Surprisingly, all strains in both groups were sensitive to both imipenem and meropenem. In group A, only one strain was resistant to ertapenem, while all strains in group B were sensitive to ertapenem. Additionally, all strains in both groups were susceptible to amikacin and tigecycline. One of the main risk factors for the development of resistance is the unregulated use of antibiotics, so the high susceptibility of our isolates to carbapenems may be in part attributed to the fact that the use of carbapenems is more restricted than the use of extended spectrum cephalosporins in Egypt.

MDR bacteria are defined as bacteria resistant to at least one antimicrobial agent from at least three different antimicrobial categories [19]. In this study, 34 out of the total 70 (48.57%) *E. coli* isolates were MDR; of which 17 (50%) MDR isolates belonged to Group A and another 17 (50%) belonged to Group B. Baljin et al. [7] reported that the fecal carriage of MDR Gram-negative bacteria, in their study, ranged from 42.2% to 69.2%, while in another study, Huang et al. [20] found that MDR *E. coli* strains accounted for 37% of the isolates in the study. A higher resistance rate was reported in another study in Bangladesh, the presence of MDR *E. coli* among fecal *E. coli* resistant to the third-generation cephalosporin was 77% [21].

Using phenotypic methods, 30 out of our 70 (42.86%) *E. coli* isolates were ESBL producers. Fourteen out of the 35 (40%) *E. coli* isolates collected at time of hospital admission were ESBL producers. After hospital admission, ten of these *E. coli* isolates remained ESBL producers, while another six isolates became ESBL producers, so that the total number of ESBL-producing isolates in group B (after hospital admission) became 16 out of the 35 (45.7%). In a study conducted by Hegal et al. [22] 12.7% of patients were colonized with ESBL-producing Enterobacteriaceae on hospital admission, while on discharge 19.6% were ESBL-producing isolates with 8.1% newly charged ESBL- producing Enterobacteriaceae.

In the present study, all ESBL-producing *E. coli* in both groups were MDR. Similarly, in a study in France, Janvier et al. reported that all their Gram-negative ESBL isolates were MDR [23]. However, in an earlier study conducted in Egypt, Abdallah et al. reported that only 47.83% of the ESBL positive Gram-negative isolates were MDR [24].

Horizontal transfer of resistance genes, which are carried on plasmids, is considered one of the main reasons for the emergence of MDR isolates [25]. In fact, the more plasmids harbored by the bacteria, the more likely it is to be resistant to antibiotics. Sharma et al. reported that strains, which, simultaneously, possessed multiple plasmids showed co-resistance to antibacterial agents from different classes [26].

In the present study, most of the *E. coli* isolates which showed multiple drug resistance were also found to harbor plasmids with molecular sizes ranging from one kb to ten kb.

Genotypically, *bla*_CTX-M_ was the most commonly detected gene among both group A and group B isolates with 71.43% and 87.50%, respectively. *bla*_TEM_ was detected in 42.86% of group A isolates and 62.5% of group B isolates, while *bla*_SHV_ was the least detected among the isolates of both group A and B with 21.43% and 6.25%, respectively.

Baljin et al. reported that, *bla*_CTX-M_ was the most abundant gene found in *E. coli* on admission to the hospital and remained the most abundant as well after 14 days [7]. Wu et al. reported that among 14 isolates with the ESBL-production *E. coli*, six isolates had *bla*_TEM_, six had *bla*_SHV_ and four had *bla*_CTX-M_ [27].

Among our isolates, two isolates harbored three genes *bla*_SHV_, *bla*_TEM_ & *bla*_CTX-M_. Eleven isolates harbored two genes *bla*_CTX-M_ and *bla*_TEM,_ while two isolates harbored *bla*_SHV_ and *bla*_TEM_. Twelve isolates harbored one gene only. In Southern Taiwan, five isolates had more than one ESBL gene [27].

Based on the genotypic ESBL detection, we report that among our 35 patients enrolled in this study, ten patients were found to carry commensal ESBL-producing *E. coli*, in two patients these isolates carried ESBL genes that were identical on admission and on discharge. However, in eight patients, these isolates carried different ESBL genes that were newly harbored during hospital stay. The high abundance of MDR commensal *E. coli* 48.57% together with the presence of 42.86% ESBL-producing commensal *E. coli* among our isolates poses an alarming threat, as they are normally associated with the increased risk of infection by the same strain, higher costs and longer hospital stay, which necessitates firm adherence to infection control measures together with strict control over antibiotics overuse.
